# Proarrhythmia associated with antiarrhythmic drugs: a comprehensive disproportionality analysis of the FDA adverse event reporting system

**DOI:** 10.3389/fphar.2023.1170039

**Published:** 2023-05-12

**Authors:** Feifei Wang, Bingfeng Zhou, Hongwei Sun, Xinan Wu

**Affiliations:** ^1^ Department of Pharmacy, Hefei BOE Hospital, Hefei, China; ^2^ Department of Cardiology, Hefei BOE Hospital, Hefei, China

**Keywords:** antiarrhythmic drugs, arrhythmia, adverse event reporting system, AAD, ventricular arrhythmia

## Abstract

**Objective:** This study aimed to identify the different associations between antiarrhythmic drugs (AADs) and arrhythmias, and to determine whether pharmacokinetic drug interactions involving AADs increase the risk of AAD-related arrhythmias compared to using AADs alone.

**Materials and methods:** The disproportionality analysis of AAD-associated cardiac arrhythmias, including AAD monotherapies and concomitant use of pharmacokinetic interacting agents involving AADs, was conducted by using reporting odds ratio (ROR) and information component (IC) as detection of potential safety signals based on FAERS data from January 2016 to June 2022. We compared the clinical features of patients reported with AAD–associated arrhythmias between fatal and non-fatal groups, and further investigated the onset time (TTO) following different AAD regimens.

**Results:** A total of 11754 AAD–associated cardiac arrhythmias reports were identified, which was more likely to occur in the elderly (52.17%). Significant signals were detected between cardiac arrhythmia and all AAD monotherapies, with ROR ranging from 4.86 with mexiletine to 11.07 with flecainide. Regarding four specific arrhythmias in High Level Term (HLT) level, the AAD monotherapies with the highest ROR were flecainide in cardiac conduction disorders (ROR025 = 21.18), propafenone in rate and rhythm disorders (ROR025 = 10.36), dofetilide in supraventricular arrhythmias (ROR025 = 17.61), and ibutilide in ventricular arrhythmias (ROR025 = 4.91). Dofetilide/ibutilide, ibutilide, mexiletine/ibutilide and dronedarone presented no signal in the above four specific arrhythmias respectively. Compared with amiodarone monotherapy, sofosbuvir plus amiodarone detected the most significantly increased ROR in arrhythmias.

**Conclusion:** The investigation showed the spectrum and risk of AAD–associated cardiac arrhythmias varied among different AAD therapies. The early identification and management of AAD-associated arrhythmias are of great importance in clinical practice.

## Introduction

Antiarrhythmic drugs (AADs) are prescribed to treat symptomatic or life-threatening arrhythmias, such as supraventricular arrhythmias and ventricular arrhythmias (Al-Khatib et al., 2018; January et al., 2019; Viskin et al., 2019; Andrade et al., 2022). Although most AADs used for treating arrhythmia have been available for decades, there is still a significant knowledge gaps in their comparative safety.

The proarrhythmic effect of AADs is a significant concern in using them (Reimold and Reynolds, 2018), which had not been systematically studied and only limited numbers of arrhythmias involving AADs were captured in clinical trials and incidental reports (Hindricks et al., 2021; Wharton et al., 2022). Despite the type of proarrhythmic events reported in previous clinical trials and meta-analyses differed among AAD treatments (Freemantle et al., 2011; Friberg, 2018; Valembois et al., 2019; Hindricks et al., 2021; Wharton et al., 2022; Singh et al., 2023), it is almost impossible to reach definitive conclusions from these studies on whether one AAD is more likely than another to result in a higher incidence of arrhythmias. The American Heart Association released a scientific statement for clinical evaluation of drug-induced arrhythmias (Tisdale et al., 2020), which have not systematically focused on the incidence of many general and specific AAD-induced arrhythmias. Moreover, drug-drug interaction (DDI) has been reported to affect the safety of AAD use, resulting in new or recurrent arrhythmias and other adverse events (Haddad and Anderson, 2002; Rajpurohit et al., 2014; Back and Burger, 2015; Mar et al., 2022). The concomitant use of amiodarone with sofosbuvir had been reported to cause serious cases of bradycardia, which may be due to sofosbuvir-based treatments displacing amiodarone from plasma binding proteins and potentiating the bradycardic effects of amiodarone (Back and Burger, 2015; Mar et al., 2022). Additionally, case reports suggested concomitant administration of flecainide with CYP2D6 inhibitors like venlafaxine and citalopram caused serious arrhythmias (Garcia, 2008; Rajpurohit et al., 2014). It is still unclear whether subsequent alterations in plasma AAD concentrations due to drug-drug interaction (DDI), compared with AAD monotherapies, can increase reporting of arrhythmias. In addition, the overviewed relationship between AADs and arrhythmias, factors related to death, potential signal spectra, as well as clinical information of AAD–related arrhythmias are still unknown.

Therefore, post-marketing surveillance is important to mine and reflect profiles of arrhythmias caused by different AAD regimens. In this study, we leveraged the Food and Drug Administration Adverse Event Reporting System (FAERS) to comprehensively characterize and investigate arrhythmias associated with AAD monotherapy and combination.

## Methods

### Data source

To investigate the association between cardiac arrhythmias and AADs, we used the FAERS database containing spontaneous adverse event reports between 1 January 2016, and 30 July 2022 to perform a disproportionality analysis. The AADs in the study included quinidine, disopyramide, mexiletine, flecainide, propafenone, sotalol, dofetilide, amiodarone, dronedarone, ibutilide, ivabradine, and adenosine. To our knowledge, pharmacokinetic drug interactions involving AADs increased the plasma concentration of AADs (Mar et al., 2022). Thus, the following concomitant use of pharmacokinetic interacting agents involving AADs were also considered in our studies: fluoxetine plus flecainide, duloxetine plus flecainide, paroxetine plus flecainide, amiodarone plus flecainide, citalopram plus propafenone, venlafaxine plus propafenone, sofosbuvir plus amiodarone, verapamil plus dronedarone, diltiazem plus dronedarone, verapamil plus ivabradine, and amiodarone plus ivabradine. Meanwhile, we searched for all adverse event reports related to concomitant use of AAD with pharmacokinetic interacting agents mentioned above. Open Vigil FDA, a pharmacovigilance tool, was adapted to extract FAERS data (Bohm et al., 2021).

### Procedures

Based on Medical Dictionary for Drug Regulatory Activities (MedDRA version 23.0), the high-level group term (HLGT) we researched was “Cardiac arrhythmias (10007521).” The full list of preferred terms (PTs) within considered cardiac arrhythmias was provided in Supplementary Table S1. The above PT level adverse events (AEs) belonged to the following four High Level Terms (HLTs): “Supraventricular arrhythmias (10042600),” “Rate and rhythm disorders NEC (10037908),” “Cardiac conduction disorders (10000032),” and “Ventricular arrhythmias and cardiac arrest (10047283).” Moreover, we collected clinical and demographic features of AE cases when data was available, including drug information (indication, concurrent medications, receipt date, treatment start and end dates), patient characteristics (gender, age, country of origin), and final patient outcomes (symptoms, seriousness). Clinical characteristics of patients with AAD-associated arrhythmias were compared between fatal and non-fatal groups. The fatal group referred to patients whose final outcome was death. The monotherapy of AAD-associated cardiac arrhythmias was defined as AAD as a primary suspected (PS) drug, without another AAD and pharmacokinetic interacting agent listed as concomitant, interacting or second suspected drugs.

### Statistical analysis

Descriptive statistics were utilized to present the clinical characteristics of the cardiac arrhythmias associated with AADs. The chi-square test was used to compare the categorical variables between the fatal and non-fatal group. We used the *t*-test and non-parametric tests (Kruskal–Wallis tests) to compare the onset time of AAD-related cardiac arrhythmias. Disproportionality analysis was conducted by using reporting odds ratio (ROR) and information component (IC) as detection of potential safety signals for AEs in the FAERS (Noren et al., 2013; Zhai et al., 2019). If there were at least three reports and one algorithm are positive, it was defined as a significant signal. All the data analysis was performed by SPSS 24.0 (SPSS Inc., Chicago, IL, United States), and *p* values <0.05 were considered significant.

## Results

### Descriptive analysis

The FAERS database recorded 70,100 AAD-associated adverse events (AEs) and 177,896 reports related to cardiac arrhythmias between January 2016 and June 2022. We identified 11754 cases of AAD-related arrhythmias and described the clinical features of reports in Table 1. The AAD-related cardiac arrhythmia AE records were mainly from the North America (5264, 44.78%) and Europe (5252, 44.68%). Regarding cardiac arrhythmia AEs, the proportion of males is greater than that of females (47.35% vs. 44.58%). Amiodarone monotherapy generated the highest number of cases related with arrhythmias (5657, 48.13%), followed by flecainide monotherapy (1675, 14.25%), and sotalol (1179, 10.03%).

**TABLE 1:** Characteristics of patients with AAD-associated cardiac arrhythmias sourced from the FAERS database (January 2016 to June 2022).

Characteristics		Total reports, *n* (%)	Fatal cases, *n* (%)	Non-fatal cases, *n* (%)	*p-value*
Total		11754	2,673	9,081	
Patient age (year)	—				*NS*
	Median (IQR)	70 (59–78)	69 (57–78)	70 (60–78)	
	<18	301 (2.56%)	82 (3.07%)	219 (2.41%)	
	18–64	3,046 (25.91%)	792 (29.63%)	2,254 (24.82%)	
	65–74	2,777 (23.63%)	586 (21.92%)	2,191 (24.13%)	
	≥75	3,355 (28.54%)	801 (29.97%)	2,554 (28.12%)	
	Unknown	2,275 (19.36%)	412 (15.41%)	1863 (20.52%)	
Gender	—				*NS*
	Female	5,240 (44.58%)	1,148 (42.95%)	4,092 (45.06%)	
	Male	5,565 (47.35%)	1,317 (49.27%)	4,248 (46.78%)	
	Unknown	949 (8.07%)	208 (7.78%)	741 (8.16%)	
Reporting year	—				*p < 0.001*
	2016	1,325 (11.27%)	153 (5.72%)	1,172 (12.91%)	
	2017	1,469 (12.50%)	204 (7.63%)	1,265 (13.93%)	
	2018	2,175 (18.50%)	294 (11.00%)	1881 (20.71%)	
	2019	2015 (17.14%)	284 (10.62%)	1731 (19.06%)	
	2020	2094 (17.82%)	267 (9.99%)	1827 (20.12%)	
	2021	1709 (14.54%)	588 (22.00%)	1,121 (12.34%)	
	2022	967 (8.23%)	883 (33.03%)	84 (0.93%)	
Area	—				*NS*
	Africa	53 (0.45%)	16 (0.60%)	37 (1.38%)	
	Asian	662 (5.63%)	185 (6.92%)	477 (17.85%)	
	Europe	5,252 (44.68%)	1,075 (40.22%)	4,177 (156.27%)	
	North America	5,264 (44.78%)	1,224 (45.79%)	4,040 (151.14%)	
	Oceania	125 (1.06%)	24 (0.90%)	101 (3.78%)	
	South America	213 (1.81%)	40 (1.50%)	173 (6.47%)	
	Unknown	185 (1.57%)	109 (4.08%)	76 (2.84%)	
Reporters	—				*NS*
	Physician	3,849 (32.75%)	997 (37.30%)	2,852 (31.41%)	
	Pharmacist	1,095 (9.32%)	252 (9.43%)	843 (9.28%)	
	Other health–professional	3,779 (32.15%)	838 (31.35%)	2,941 (32.39%)	
	Consumer or Non–health professional	2,818 (23.97%)	555 (20.76%)	2,263 (24.92%)	
	Unknown	213 (1.81%)	31 (1.16%)	182 (2.00%)	
AAD as suspected drug	—				*NS*
	Monotherapy	11344 (96.51%)	2,587 (96.78%)	8,757 (96.43%)	*NS*
	Quinidine	27 (0.23%)	7 (0.26%)	20 (0.22%)	
	Disopyramide	54 (0.46%)	14 (0.52%)	40 (0.44%)	
	Mexiletine	65 (0.55%)	19 (0.71%)	46 (0.51%)	
	Flecainide	1,675 (14.25%)	346 (12.94%)	1,329 (14.63%)	
	Propafenone	644 (5.48%)	86 (3.22%)	558 (6.14%)	
	Sotalol	1,179 (10.03%)	225 (8.42%)	954 (10.51%)	
	Dofetilide	778 (6.62%)	96 (3.59%)	682 (7.51%)	
	Amiodarone	5,657 (48.13%)	1,502 (56.19%)	4,155 (45.75%)	
	Dronedarone	379 (3.22%)	46 (1.72%)	333 (3.67%)	
	Ibutilide	6 (0.05%)	1 (0.04%)	5 (0.06%)	
	Ivabradine	757 (6.44%)	212 (7.93%)	545 (6.00%)	
	Adenosine	123 (1.05%)	33 (1.23%)	90 (0.99%)	
	Combination therapy	410 (3.49%)	86 (3.22%)	324 (3.57%)	*NS*
	Fluoxetine + Flecainide	29 (0.25%)	10 (0.37%)	19 (0.21%)	
	Duloxetine + Flecainide	39 (0.33%)	6 (0.22%)	33 (0.36%)	
	Paroxetine + Flecainide	20 (0.17%)	2 (0.07%)	18 (0.20%)	
	Amiodarone + Flecainide	95 (0.81%)	35 (1.31%)	60 (0.66%)	
	Citalopram + Propafenone	17 (0.14%)	3 (0.11%)	14 (0.15%)	
	Venlafaxine + Propafenone	20 (0.17%)	2 (0.07%)	18 (0.20%)	
	Sofosbuvir + Amiodarone	66 (0.56%)	6 (0.22%)	60 (0.66%)	
	Verapamil + Dronedarone	3 (0.03%)	0 (0.00%)	3 (0.03%)	
	Diltiazem + Dronedarone	27 (0.23%)	4 (0.15%)	23 (0.25%)	
	Verapamil + Ivabradine	18 (0.15%)	2 (0.07%)	16 (0.18%)	
	Amiodarone + Ivabradine	76 (0.65%)	16 (0.60%)	60 (0.66%)	

Abbreviations: FAERS, Food and Drug Administration’s Adverse Event Reporting System; IQR, interquartile range; N, number of records; AAD, antiarrhythmic drug; *p* values was calculated by the chi-square test.

As shown in Table 1, no significant difference was found in patient gender, age, area, reporter and AAD regimen for fatal vs. non-fatal reports.

### Signal values related to different AAD regimens

The signal values and the association between AADs and arrhythmias were shown in Table 2. All studied AAD monotherapies were significantly correlated with the reporting frequency of cardiac arrhythmia (HLGT), with ROR ranging from 4.86 with mexiletine to 11.07 with flecainide (Table 2). Regarding four specific arrhythmias in HLT level, the AAD monotherapies with the highest ROR were flecainide in cardiac conduction disorders (ROR025 = 23.22), propafenone in rate and rhythm disorders (ROR025 = 11.32), dofetilide in supraventricular arrhythmias (ROR025 = 18.85), and ibutilide in ventricular arrhythmias (ROR025 = 11.47). Dofetilide/ibutilide, ibutilide, mexiletine/ibutilide and dronedarone presented no signal in the above four specific arrhythmias respectively. Compared with amiodarone monotherapy, sofosbuvir plus amiodarone detected the most significantly increased ROR in arrhythmias. Four in eleven different class-specific AAD combination therapy (amiodarone plus flecainide, sofosbuvir plus amiodarone, verapamil plus ivabradine, and amiodarone plus ivabradine) were detected with pharmacovigilance signals of cardiac arrhythmias (HLGT) compared with AAD monotherapy.

**TABLE 2:** Associations of different AAD regimens with cardiac arrhythmias in HLGT and HLT level.

Strategy	Drug	Arrhythmias		Cardiac conduction disorders		Rate and rhythm disorders NEC		Supraventricular arrhythmias		Ventricular arrhythmias and cardiac arrest	
		N	OR (95% CI)	N	OR (95% CI)	N	OR (95% CI)	N	OR (95% CI)	N	OR (95% CI)
Total	Antiarrhythmic Drugs	11754	8.53 (8.41–8.66)	1,101	12.02 (11.5–12.57)	4,241	7.49 (7.32–7.66)	5,126	12.81 (12.54–13.09)	3,474	8.18 (7.98–8.39)
Monotherapy	Quinidine	27	5.03 (3.78–6.69)	12	15.08 (9.96–22.82)	7	2.95 (1.72–5.05)	7	3.86 (2.26–6.60)	10	5.17 (3.29–8.12)
	Disopyramide	54	5.55 (4.54–6.8)	3	3.14 (1.39–7.06)	19	4.51 (3.25–6.26)	16	5.15 (3.60–7.36)	26	7.87 (5.93–10.44)
	Mexiletine	65	4.86 (4.05–5.84)	5	4.05 (2.16–7.59)	17	3.00 (2.13–4.24)	6	1.52 (0.86–2.71)	48	10.80 (8.75–13.32)
	Flecainide	1,675	11.07 (10.65–11.51)	243	23.22 (21.18–25.46)	664	10.63 (10.04–11.26)	752	16.99 (16.09–17.93)	396	8.44 (7.85–9.08)
	Propafenone	644	10.89 (10.24–11.6)	71	16.32 (13.78–19.31)	278	11.32 (10.36–12.36)	305	17.43 (16.01–18.98)	159	8.59 (7.66–9.63)
	Sotalol	1,179	7.60 (7.27–7.94)	82	7.68 (6.57–8.98)	394	6.15 (5.72–6.62)	556	12.25 (11.52–13.04)	296	6.16 (5.67–6.69)
	Dofetilide	778	9.01 (8.52–9.52)	8	1.37 (0.84–2.26)	188	5.26 (4.74–5.84)	479	18.85 (17.61–20.18)	198	7.36 (6.65–8.15)
	Amiodarone	5,657	8.16 (7.99–8.33)	503	10.86 (10.19–11.58)	1996	7.00 (6.78–7.23)	2,297	11.40 (11.05–11.76)	1899	8.88 (8.59–9.19)
	Dronedarone	379	7.69 (7.11–8.31)	21	5.76 (4.24–7.83)	123	6.00 (5.27–6.83)	254	17.34 (15.80–19.02)	19	1.25 (0.91–1.73)
	Ibutilide	6	9.08 (3.89–21.19)	1	—	0	—	1	—	6	11.47 (4.91–26.78)
	Ivabradine	757	6.99 (6.62–7.39)	72	9.49 (8.04–11.21)	350	7.82 (7.24–8.45)	234	7.37 (6.71–8.10)	222	6.60 (6.00–7.27)
	Adenosine	123	6.55 (5.72–7.5)	24	14.29 (10.70–19.10)	34	4.29 (3.36–5.48)	51	8.84 (7.23–10.82)	52	8.54 (6.99–10.42)
	Fluoxetine + Flecainide vs. Flecainide	29	1.15 (0.85–1.56)	8	2.05 (1.22–3.46)	13	1.29 (0.85–1.96)	13	1.15 (0.76–1.74)	14	2.26 (1.50–3.38)
	Duloxetine + Flecainide vs. Flecainide	39	0.87 (0.68–1.12)	9	1.36 (0.84–2.21)	18	1.02 (0.72–1.44)	16	0.80 (0.55–1.16)	20	1.85 (1.32–2.60)
	Paroxetine + Flecainide vs. Flecainide	20	0.71 (0.50–0.99)	1	—	15	1.31 (0.89–1.93)	0	—	6	0.90 (0.50–1.62)
	Amiodarone + Flecainide vs. Flecainide	95	1.29 (1.08–1.54)	8	0.76 (0.46–1.27)	36	1.23 (0.96–1.59)	32	0.97 (0.74–1.27)	40	2.27 (1.77–2.90)
	Citalopram + Propafenone vs. Propafenone	17	0.79 (0.54–1.16)	2	—	5	0.56 (0.29–1.07)	7	0.70 (0.40–1.22)	5	0.94 (0.49–1.81)
	Venlafaxine + Propafenone vs. Propafenone	20	1.23 (0.85–1.78)	0	—	18	2.47 (1.68–3.65)	1	—	2	—
	Sofosbuvir + Amiodarone vs. Amiodarone	66	4.10 (3.03–5.55)	16	8.69 (5.90–12.81)	35	5.87 (4.36–7.90)	31	4.58 (3.37–6.21)	18	3.20 (2.22–4.62)
	Verapamil + Dronedarone vs. Dronedarone	3	0.95 (0.35–2.00)	0	—	1	—	2	—	0	—
	Diltiazem + Dronedarone vs. Dronedarone	27	1.96 (0.70–1.29)	1	—	4	0.46 (0.23–0.95)	23	1.21 (0.87–1.67)	1	—
	Verapamil + Ivabradine vs. Ivabradine	18	1.96 (1.32–2.89)	2	—	7	1.62 (0.92–2.84)	11	3.52 (2.19–5.64)	1	—
	Amiodarone + Ivabradine vs. Ivabradine	76	2.01 (1.65–2.45)	9	2.34 (1.41–3.88)	19	1.09 (0.78–1.54)	32	2.69 (2.03–3.56)	36	3.17 (2.42–4.16)

Abbreviations: HLGT, high-level group term; HLT, high level term; N: number of records; ROR025, the lower end of the 95% confidence interval of ROR; ROR975, the upper end of the 95% confidence interval of IC; IC025, the lower end of the 95% confidence interval of IC; *p* values was calculated by the chi-square test.

### The signal spectrum of cardiac arrhythmias differs in AAD strategies

The arrhythmia signal spectra of different AAD therapies were shown in Table 3. Amiodarone presented a broadest spectrum of cardiac arrhythmias AEs, with 42 PTs detected as positive signals, ranging from cardiac flutter (IC 025 = 0.72) to torsade de pointes (TdP) (IC 025 = 4.93). There were 38 PTs as signals associated with flecainide, with signal values ranging from IC 025 = 1.08 (long QT syndrome) to IC 025 = 4.88 (atrioventricular block first degree). However, the drug with the least PTs was ibutilide, with only one signal detected, followed by quinidine, with five signals detected. Ventricular tachycardia, ventricular fibrillation and atrial fibrillation were three overlapping PTs, all of which were found significantly associated with disopyramide, flecainide, propafenone, sotalol, dofetilide, amiodarone, ivabradine, and adenosine. Torsade de pointes were detected as the strongest signal in amiodarone (IC 025 = 4.93).

**TABLE 3:** Arrhythmia signal profiles of different AAD strategies.

PT	Atrioventricular block complete	—	—	—	3.31	3.33	1.41	—	3.15	0.77	—	1.64	2.07
	Atrioventricular block first degree	—	—	—	4.88	3.71	1.47	—	2.75	—	—	−0.08	—
	Brugada syndrome	0.71	—	—	4.45	0.43	—	—	1.17	—	—	—	—
	Bundle branch block right	—	—	—	3.90	2.39	0.53	—	2.23	—	—	1.46	—
	Defect conduction intraventricular	—	—	—	4.23	1.78	—	—	0.89	—	—	—	—
	Atrioventricular block	3.12	—	—	2.68	2.21	2.12	−1.10	3.17	2.37	—	2.01	3.33
	Atrioventricular block second degree	—	—	—	2.10	—	1.89	—	2.11	2.70	—	2.20	—
	Bundle branch block	—	—	—	2.54	—	—	—	2.91	—	—	—	—
	Bundle branch block left	—	—	—	3.32	2.87	−1.04	—	2.95	1.08	—	3.43	0.37
	Conduction disorder	—	—	—	3.69	2.40	—	—	1.82	—	—	1.50	—
	Long QT syndrome	—	—	1.68	1.08	—	2.76	—	3.67	—	—	—	—
	Sinoatrial block	—	—	—	3.29	—	—	—	2.71	—	—	—	—
	Arrhythmia	0.44	1.63	1.31	3.58	3.58	2.92	3.21	2.68	2.75	—	1.58	−1.43
	Bradyarrhythmia	—	—	—	4.08	2.85	—	—	3.02	2.20	—	—	—
	BRASH syndrome	—	—	—	—	—	3.05	—	4.26	—	—	—	—
	Cardiac flutter	—	—	—	2.81	2.60	1.84	2.24	0.72	1.67	—	—	—
	Tachyarrhythmia	—	—	—	4.62	0.72	—	0.49	4.59	—	—	1.20	—
	Bradycardia	0.31	1.22	—	3.81	3.83	3.00	−0.29	3.63	1.71	—	3.67	1.43
	Cardiac fibrillation	—	—	—	1.39	2.19	0.49	0.16	1.65	0.38	—	0.71	—
	Extrasystoles	—	—	—	1.77	2.47	0.76	2.71	1.99	0.70	—	1.17	—
	Tachycardia	—	—	−0.61	1.99	1.34	0.69	1.19	1.21	0.98	—	1.97	0.57
	Arrhythmia supraventricular	—	—	—	1.98	1.62	0.10	1.01	2.27	—	—	1.81	—
	Atrial fibrillation	—	1.56	−1.91	4.04	3.84	3.65	4.40	3.50	4.27	—	1.48	1.34
	Nodal arrhythmia	—	—	—	1.44	3.12	—	—	3.48	—	—	—	—
	Sinus arrest	—	—	—	1.74	1.89	1.72	—	1.56	—	—	2.44	0.60
	Sinus bradycardia	—	—	—	3.28	4.09	2.61	0.21	3.66	−0.76	—	3.85	—
	Sinus tachycardia	—	—	—	−0.01	—	−1.26	—	−0.67	—	—	3.03	−0.22
	Atrial flutter	—	—	—	4.53	3.7	3.76	3.69	3.94	3.12	—	1.12	—
	Atrial tachycardia	—	—	—	2.81	—	2.09	1.95	4.41	—	—	4.08	—
	Nodal rhythm	—	—	—	0.00	1.07	—	—	2.50	—	—	—	—
	Sinus arrhythmia	—	—	—	—	—	—	1.49	1.65	0.49	—	—	—
	Sinus node dysfunction	—	—	—	3.50	—	1.50	0.98	4.05	0.15	—	2.73	—
	Supraventricular extrasystoles	—	—	—	2.14	−1.81	1.08	2.77	3.28	—	—	3.21	—
	Supraventricular tachycardia	—	—	—	3.06	2.61	1.16	1.34	1.81	—	—	0.65	4.50
	Cardiac arrest	—	1.74	−1.55	2.66	2.36	1.37	2.00	2.24	−1.68	—	1.29	1.26
	Sudden death	—	—	—	—	—	0.65	—	1.58	—	—	2.45	—
	Ventricular arrhythmia	—	0.52	2.81	2.73	—	2.71	0.07	3.73	—	—	3.16	—
	Ventricular extrasystoles	—	—	2.60	2.14	—	1.08	2.77	3.28	0.04	—	3.21	0.73
	Ventricular tachycardia	1.05	1.29	3.90	4.35	4.12	3.49	3.92	4.57	−1.17	—	3.43	3.39
	Cardio-respiratory arrest	—	0.62	—	1.98	2.45	1.63	−1.92	2.10	—	—	1.64	−1.19
	Pulseless electrical activity	—	—	—	3.59	0.26	—	—	1.92	—	—	1.19	—
	Torsade de pointes	—	—	1.80	3.31	—	4.84	4.51	4.93	—	1.89	4.35	—
	Ventricular fibrillation	—	2.18	1.33	3.93	2.00	1.85	3.07	4.34	−0.75	—	3.26	3.67
	IC025 ≤ 0	Quinidine	Disopyramide	Mexiletine	Flecainide	Propafenone	Sotalol	Dofetilide	Amiodarone	Dronedarone	Ibutilide	Ivabradine	Adenosine
	4> IC025 > 0												
	IC025 ≥ 4												

### Time to onset of AAD–Associated cardiac arrhythmia adverse effects

A total of 3742 AAD–associated cardiac arrhythmias reported the time to onset (TTO), as shown in Table 4. (There were no or few known data on quinidine and ibutilide, which was not shown in Table 4). According to all AADs, the median onset time is 45 days, and the interquartile range is 3–331 days. Among AAD monotherapies, we found significant differences in the reported TTO of arrhythmias (*p < 0.001*). The median TTO was 46 days for amiodarone (IQR 5-330), 47 days for flecainide (IQR 4-349), 112 days for propafenone (IQR 3-433), 165 days for dronedarone (IQR 14-565), 64 days for sotalol (IQR 3-351), 13 days for disopyramide (IQR 0-84), 0 days for ibutilide (IQR 0-0), 14 days for ivabradine (IQR 0-132), 65 days for adenosine (IQR 0-366), 43 days for dofetilide (IQR 2-332), and 11 days for mexiletine (IQR 1-139), respectively. Moreover, there was no significant difference in the TTO between AAD monoregimen and combinationtherapy (flecainide vs. fluoxetine/duloxetine/paroxetine/amiodarone plus flecainide, *p* = 0.117; propafenone vs. citalopram/venlafaxine plus propafenone, *p* = 0.525; amiodarone vs. sofosbuvir plus amiodarone, *p* = 0.061; dronedarone vs. verapamil/diltiazem plus dronedarone, *p* = 0.411; dronedarone vs. verapamil/diltiazem plus dronedarone, *p* = 0.525; ivabradine vs. verapamil/amiodarone plus ivabradine, *p* = 0.444).

**TABLE 4:** Onset time of AADs–associated arrhythmias.

	Median (IQR)	0–30	31–60	61–90	91–120	121–180	181–360	Greater than 360	Unknown
Quinidine (*n* = 27)	--	0 (0.00%)	0 (0.00%)	0 (0.00%)	0 (0.00%)	0 (0.00%)	0 (0.00%)	0 (0.00%)	27 (100.00%)
Disopyramide (*n* = 54)	13 (0–84)	10 (18.52%)	1 (1.85%)	0 (0.00%)	0 (0.00%)	0 (0.00%)	1 (1.85%)	2 (3.70%)	40 (74.07%)
Mexiletine (*n* = 65)	11 (1–139)	12 (18.46%)	0 (0.00%)	1 (1.54%)	1 (1.54%)	1 (1.54%)	1 (1.54%)	3 (4.62%)	46 (70.77%)
Flecainide (*n* = 1,675)	47 (4–349)	216 (12.90%)	37 (2.21%)	23 (1.37%)	10 (0.60%)	21 (1.25%)	50 (2.99%)	118 (7.04%)	1,200 (71.64%)
Propafenone (*n* = 644)	112 (3–433)	63 (9.78%)	7 (1.09%)	5 (0.78%)	10 (1.55%)	9 (1.40%)	20 (3.11%)	47 (7.30%)	483 (75.00%)
Sotalol (*n* = 1,179)	64 (3–351)	181 (15.35%)	22 (1.87%)	29 (2.46%)	22 (1.87%)	16 (1.36%)	41 (3.48%)	103 (8.74%)	765 (64.89%)
Dofetilide (*n* = 778)	43 (2–332)	128 (16.45%)	18 (2.31%)	11 (1.41%)	12 (1.54%)	15 (1.93%)	23 (2.96%)	66 (8.48%)	505 (64.91%)
Amiodarone (*n* = 5,657)	46 (5–330)	864 (15.27%)	141 (2.49%)	97 (1.71%)	38 (0.67%)	101 (1.79%)	219 (3.87%)	446 (7.88%)	3,751 (66.31%)
Dronedarone (*n* = 379)	165 (14–565)	29 (7.65%)	9 (2.37%)	3 (0.79%)	3 (0.79%)	8 (2.11%)	11 (2.90%)	36 (9.50%)	280 (73.89%)
Ibutilide (*n* = 6)	0 (0–0)	2 (33.33%)	0 (0.00%)	0 (0.00%)	0 (0.00%)	0 (0.00%)	0 (0.00%)	0 (0.00%)	4 (66.67%)
Ivabradine (*n* = 757)	14 (0–132)	135 (17.83%)	17 (2.25%)	13 (1.72%)	9 (1.19%)	13 (1.72%)	12 (1.59%)	39 (5.15%)	519 (68.56%)
Adenosine (*n* = 123)	65 (0–366)	11 (8.94%)	1 (0.81%)	4 (3.25%)	1 (0.81%)	2 (1.63%)	1 (0.81%)	6 (4.88%)	97 (78.86%)
Fluoxetine/Duloxetine/Paroxetine/Amiodarone + Flecainide (*n* = 183)	18 (2–245)	26 (14.21%)	1 (0.55%)	1 (0.55%)	0 (0.00%)	2 (1.09%)	5 (2.73%)	7 (3.83%)	140 (76.50%)
Citalopram/Venlafaxine + Propafenone (*n* = 37)	19 (12–474)	5 (13.51%)	1 (2.70%)	0 (0.00%)	0 (0.00%)	0 (0.00%)	0 (0.00%)	3 (8.11%)	28 (75.68%)
Sofosbuvir + Amiodarone (*n* = 66)	18 (0–81)	12 (18.18%)	2 (3.03%)	4 (6.06%)	0 (0.00%)	0 (0.00%)	0 (0.00%)	5 (7.58%)	43 (65.15%)
Verapamil/Diltiazem + Dronedarone (*n* = 30)	325 (1–668)	3 (10.00%)	1 (3.33%)	0 (0.00%)	0 (0.00%)	0 (0.00%)	2 (6.67%)	5 (16.67%)	19 (63.33%)
Verapamil/Amiodarone + Ivabradine (*n* = 94)	0 (0–146)	18 (19.15%)	1 (1.06%)	1 (1.06%)	1 (1.06%)	2 (2.13%)	1 (1.06%)	5 (5.32%)	65 (69.15%)

Abbreviations: N, number of records; IQR, interquartile range; AAD, antiarrhythmic drug.

## Discussion

This study comprehensively evaluated the adverse events of AAD-induced cardiac arrhythmias based on the FAERS database. By employing the FAERS database, we analyzed the clinical characteristics, spectrum, TTO, and outcomes of AAD-induced arrhythmia AEs.

To assess the proarrhythmic effects of AADs, our research detected significant signals between cardiac arrhythmia and all AAD monotherapies, with ROR ranging from 4.86 with mexiletine to 11.07 with flecainide. In the disproportionate analysis of arrhythmias at HLT level, ibutilide monotherapy presented no signal in three specific arrhythmias except for ventricular arrhythmias and cardiac arrest, while mexiletine, dofetilide and dronedarone monotherapy demonstrated negative signal in supraventricular arrhythmias, cardiac conduction disorders, and ventricular arrhythmias and cardiac arrest, respectively. Notably, the risk of ventricular arrhythmia/TdP of dronedarone varied in different literatures, some of which showed a lower risk of dronedarone (Lafuente-Lafuente et al., 2012; Friberg, 2018; Tisdale et al., 2020), while others showed the opposite (Kao et al., 2012; Wu et al., 2022). Previous study reported 138 cases of ventricular arrhythmia associated with dronedarone between July 2009 and June 2011 (Kao et al., 2012), while our research identified only 19 reports during January 2016–June 2022. Additionally, the FAERS database recorded 61 cases of TdP related to dronedarone from the first quarter of 2009 to the fourth quarter of 2015 but only 2 reports between January 2016 and June 2022, resulting in the positive signal of TdP in dronedarone after incorporating data before 2016 (Kao et al., 2012; Wu et al., 2022). The higher reports of ventricular arrhythmia/TdP before 2016 and the lower cases after 2016 may be related to the early non-standard use of dronedarone, as it clearly worsens outcomes in patients with decompensated heart failure (Kober et al., 2008) and permanent atrial fibrillation (Rosenstein and Woods, 2012). The negative signal of dronedarone in ventricular arrhythmia/TdP shown in our study is updated and more consistent with clinical research and meta-analysis (Hohnloser et al., 2009; Freemantle et al., 2011; Lafuente-Lafuente et al., 2012; Friberg, 2018; Reimold and Reynolds, 2018; Valembois et al., 2019; Tisdale et al., 2020; Wharton et al., 2022), and will provide more accurate reference for the selection of AAD in clinical practice.

As compared to studied AAD monotherapy, seven pharmacokinetic drug interactions involving AADs were associated with a higher risk of reports of cardiac arrhythmias at HLGT or HLT level, which provided evidence for and endorsed the warnings included in the prescribing information of these drugs (Gareri et al., 2008; Back and Burger, 2015; McDonald et al., 2015; Tisdale, 2016; Mar et al., 2022). Four in eleven different specific AAD combination therapies (paroxetine plus flecainide vs. flecainide, diltiazem/verapamil plus dronedarone vs. dronedarone, citalopram plus propafenone vs. propafenone) were detected with no signal of cardiac arrhythmias at HLGT and HLT level compared with monotherapy, which was not affected by an increase in ADD concentrations demonstrated in previous studies (Garcia, 2008; Tisdale, 2016; Mar et al., 2022). Owing to the lack of studies on arrhythmias associated with AAD combination therapy, the rationale for no increased signal for the above four combination need to be further elucidated and explored.

Atrial fibrillation (AF) induced by disopyramide, adenosine and ivabradine was over-reported, but the signal intensity was weak; quinidine, mexiletine and ibutilide did not present a significant signal value. Ivabradine presented weak association with over-reporting frequency of AF in our study, consistent with the increased AF incidence with ivabradine found in previous clinical trials (Fox et al., 2008; Swedberg et al., 2010; Tendera et al., 2011; Fox et al., 2014; Bohm et al., 2015; Fox et al., 2015; Koruth et al., 2017). Prior studies showed that patients in the ivabradine group were more likely to develop new-onset AF (Fox et al., 2015; Koruth et al., 2017), and were associated with increased risk of AF in a previous meta-analysis (Martin et al., 2014). Moreover, the evidence concerning effect of ivabradine on AF in preclinical and clinical studies was conflicting, which provided modest evidence for ivabradine to reduce the incidence of AF in animal models (Li et al., 2015; Wang et al., 2019), but provided strong evidence for increased incidence of AF in human models by ivabradine (Fox et al., 2008; Swedberg et al., 2010; Tendera et al., 2011; Fox et al., 2014; Bohm et al., 2015); however, there is a concept that ivabradine in combination with beta-blockers could successfully control heart rate in AF, which is currently being investigated in a placebo-controlled clinical trials (RCT) (Fontenla et al., 2020). Although at risk of inducing atrial fibrillation, according to the 2021 update to the 2017 ACC expert consensus decision pathway for optimization of heart failure treatment, a history of paroxysmal AF is not a contraindication to ivabradine (Writing et al., 2021). Further studies are needed to establish the role of ivabradine in AF.

The time interval between the initial of AAD therapy to the onset of arrhythmia varies greatly. There was significant difference in the distribution of TTO among AAD mono-regimens (*p < 0.001*). According to all AADs, the median onset time is 45 days, with a interquartile range of 3–331 days, suggesting the significance of cardiac monitoring during the higher-risk time window of 45 days and individualized cardiac monitoring after AAD administration. Moreover, there was no significant difference in the TTO between AAD mono regimen and combination therapy.

Our study has certain limitations inherent to pharmacovigilance databases. Firstly, the true incidence of AE is unclear owing to the voluntary nature of FAERS reporting, including missing information, misspelled drug names, under-reporting and over-reporting, all of which are common in databases. Secondly, a slight increase of ROR only provided safety signals, not real risks of AE in clinical practice, which may be relevant and need further confirmation. Thirdly, due to the lack of denominator, we can neither calculate the incidence rate nor quantify the adverse reaction signals for AAD-related arrhythmias.

## Conclusion

We reviewed arrhythmia AEs related with AADs from the FAERS database, as well as assessing whether pharmacokinetic drug interactions involving AADs increased the risk of arrhythmias compared to using AADs alone. Our research is practical for clinicians to understand the safety profile of AADs for arrhythmia and optimize their use among individual patients.

## Data Availability

The original contributions presented in the study are included in the article/Supplementary Material, further inquiries can be directed to the corresponding authors.
