# Supplementary material for: Proarrhythmia associated with antiarrhythmic drugs: a comprehensive disproportionality analysis of the FDA adverse event reporting system
Source: Front Pharmacol. 2023 May 12;14:1170039. doi: 10.3389/fphar.2023.1170039 (PMC10213327; doi:10.3389/fphar.2023.1170039)
Supplement: Supplementary file 1 [file Table1.DOCX]

Table 1 Characteristics of patients with AAD-associated cardiac arrhythmias sourced from the FAERS database (January 2016 to June 2022).

| Characteristics |  | Total reports,  n (%) | Fatal cases,  n (%) | Non-fatal cases,  n (%) | *p-value* |
| --- | --- | --- | --- | --- | --- |
| Total |  | 11754 | 2673 | 9081 |  |
| Patient age (year) | — |  |  |  | *NS* |
|  | Median (IQR) | 70(59-78) | 69(57-78) | 70(60-78) |  |
|  | < 18 | 301(2.56%) | 82(3.07%) | 219(2.41%) |  |
|  | 18–64 | 3046(25.91%) | 792(29.63%) | 2254(24.82%) |  |
|  | 65–74 | 2777(23.63%) | 586(21.92%) | 2191(24.13%) |  |
|  | ≥75 | 3355(28.54%) | 801(29.97%) | 2554(28.12%) |  |
|  | Unknown | 2275(19.36%) | 412(15.41%) | 1863(20.52%) |  |
| Gender | — |  |  |  | *NS* |
|  | Female | 5240(44.58%) | 1148(42.95%) | 4092(45.06%) |  |
|  | Male | 5565(47.35%) | 1317(49.27%) | 4248(46.78%) |  |
|  | Unknown | 949(8.07%) | 208(7.78%) | 741(8.16%) |  |
| Reporting year | — |  |  |  | *p < 0.001* |
|  | 2016 | 1325(11.27%) | 153(5.72%) | 1172(12.91%) |  |
|  | 2017 | 1469(12.50%) | 204(7.63%) | 1265(13.93%) |  |
|  | 2018 | 2175(18.50%) | 294(11.00%) | 1881(20.71%) |  |
|  | 2019 | 2015(17.14%) | 284(10.62%) | 1731(19.06%) |  |
|  | 2020 | 2094(17.82%) | 267(9.99%) | 1827(20.12%) |  |
|  | 2021 | 1709(14.54%) | 588(22.00%) | 1121(12.34%) |  |
|  | 2022 | 967(8.23%) | 883(33.03%) | 84(0.93%) |  |
| Area | — |  |  |  | *NS* |
|  | Africa | 53(0.45%) | 16(0.60%) | 37(1.38%) |  |
|  | Asian | 662(5.63%) | 185(6.92%) | 477(17.85%) |  |
|  | Europe | 5252(44.68%) | 1075(40.22%) | 4177(156.27%) |  |
|  | North America | 5264(44.78%) | 1224(45.79%) | 4040(151.14%) |  |
|  | Oceania | 125(1.06%) | 24(0.90%) | 101(3.78%) |  |
|  | South America | 213(1.81%) | 40(1.50%) | 173(6.47%) |  |
|  | Unknown | 185(1.57%) | 109(4.08%) | 76(2.84%) |  |
| Reporters | — |  |  |  | *NS* |
|  | Physician | 3849(32.75%) | 997(37.30%) | 2852(31.41%) |  |
|  | Pharmacist | 1095(9.32%) | 252(9.43%) | 843(9.28%) |  |
|  | Other health–professional | 3779(32.15%) | 838(31.35%) | 2941(32.39%) |  |
|  | Consumer or Non–health professional | 2818(23.97%) | 555(20.76%) | 2263(24.92%) |  |
|  | Unknown | 213(1.81%) | 31(1.16%) | 182(2.00%) |  |
| AAD as  suspected drug | — |  |  |  | *NS* |
|  | Monotherapy | 11344(96.51%) | 2587(96.78%) | 8757(96.43%) | *NS* |
|  | Quinidine | 27(0.23%) | 7(0.26%) | 20(0.22%) |  |
|  | Disopyramide | 54(0.46%) | 14(0.52%) | 40(0.44%) |  |
|  | Mexiletine | 65(0.55%) | 19(0.71%) | 46(0.51%) |  |
|  | Flecainide | 1675(14.25%) | 346(12.94%) | 1329(14.63%) |  |
|  | Propafenone | 644(5.48%) | 86(3.22%) | 558(6.14%) |  |
|  | Sotalol | 1179(10.03%) | 225(8.42%) | 954(10.51%) |  |
|  | Dofetilide | 778(6.62%) | 96(3.59%) | 682(7.51%) |  |
|  | Amiodarone | 5657(48.13%) | 1502(56.19%) | 4155(45.75%) |  |
|  | Dronedarone | 379(3.22%) | 46(1.72%) | 333(3.67%) |  |
|  | Ibutilide | 6(0.05%) | 1(0.04%) | 5(0.06%) |  |
|  | Ivabradine | 757(6.44%) | 212(7.93%) | 545(6.00%) |  |
|  | Adenosine | 123(1.05%) | 33(1.23%) | 90(0.99%) |  |
|  | Combination therapy | 410(3.49%) | 86(3.22%) | 324(3.57%) | *NS* |
|  | Fluoxetine + Flecainide | 29(0.25%) | 10(0.37%) | 19(0.21%) |  |
|  | Duloxetine + Flecainide | 39(0.33%) | 6(0.22%) | 33(0.36%) |  |
|  | Paroxetine + Flecainide | 20(0.17%) | 2(0.07%) | 18(0.20%) |  |
|  | Amiodarone + Flecainide | 95(0.81%) | 35(1.31%) | 60(0.66%) |  |
|  | Citalopram + Propafenone | 17(0.14%) | 3(0.11%) | 14(0.15%) |  |
|  | Venlafaxine + Propafenone | 20(0.17%) | 2(0.07%) | 18(0.20%) |  |
|  | Sofosbuvir + Amiodarone | 66(0.56%) | 6(0.22%) | 60(0.66%) |  |
|  | Verapamil + Dronedarone | 3(0.03%) | 0(0.00%) | 3(0.03%) |  |
|  | Diltiazem + Dronedarone | 27(0.23%) | 4(0.15%) | 23(0.25%) |  |
|  | Verapamil + Ivabradine | 18(0.15%) | 2(0.07%) | 16(0.18%) |  |
|  | Amiodarone + Ivabradine | 76(0.65%) | 16(0.60%) | 60(0.66%) |  |

Abbreviations: FAERS: Food and Drug Administration's Adverse Event Reporting System; IQR: interquartile range; N: number of records; AAD：antiarrhythmic drug;  *p* values was calculated by the chi-square test.

Table 2 Associations of different AAD regimens with cardiac arrhythmias in HLGT and HLT level.

| **Strategy** | **Drug** | **Arrhythmias** | | **Cardiac conduction disorders** | | **Rate and rhythm  disorders NEC** | | **Supraventricular arrhythmias** | | **Ventricular arrhythmias and cardiac arrest** | |
| --- | --- | --- | --- | --- | --- | --- | --- | --- | --- | --- | --- |
|  |  | N | OR (95% CI) | N | OR (95% CI) | N | OR (95% CI) | N | OR (95% CI) | N | OR (95% CI) |
| Total | Antiarrhythmic Drugs | 11754 | 8.53(8.41-8.66) | 1101 | 12.02(11.5-12.57) | 4241 | 7.49(7.32-7.66) | 5126 | 12.81(12.54-13.09) | 3474 | 8.18(7.98-8.39) |
| Monotherapy | Quinidine | 27 | 5.03(3.78-6.69) | 12 | 15.08(9.96-22.82) | 7 | 2.95(1.72-5.05) | 7 | 3.86(2.26-6.60) | 10 | 5.17(3.29-8.12) |
|  | Disopyramide | 54 | 5.55(4.54-6.8) | 3 | 3.14(1.39-7.06) | 19 | 4.51(3.25-6.26) | 16 | 5.15(3.60-7.36) | 26 | 7.87(5.93-10.44) |
|  | Mexiletine | 65 | 4.86(4.05-5.84) | 5 | 4.05(2.16-7.59) | 17 | 3.00(2.13-4.24) | 6 | 1.52(0.86-2.71) | 48 | 10.80(8.75-13.32) |
|  | Flecainide | 1675 | 11.07(10.65-11.51) | 243 | 23.22(21.18-25.46) | 664 | 10.63(10.04-11.26) | 752 | 16.99(16.09-17.93) | 396 | 8.44(7.85-9.08) |
|  | Propafenone | 644 | 10.89(10.24-11.6) | 71 | 16.32(13.78-19.31) | 278 | 11.32(10.36-12.36) | 305 | 17.43(16.01-18.98) | 159 | 8.59(7.66-9.63) |
|  | Sotalol | 1179 | 7.60(7.27-7.94) | 82 | 7.68(6.57-8.98) | 394 | 6.15(5.72-6.62) | 556 | 12.25(11.52-13.04) | 296 | 6.16(5.67-6.69) |
|  | Dofetilide | 778 | 9.01(8.52-9.52) | 8 | 1.37(0.84-2.26) | 188 | 5.26(4.74-5.84) | 479 | 18.85(17.61-20.18) | 198 | 7.36(6.65-8.15) |
|  | Amiodarone | 5657 | 8.16(7.99-8.33) | 503 | 10.86(10.19-11.58) | 1996 | 7.00(6.78-7.23) | 2297 | 11.40(11.05-11.76) | 1899 | 8.88(8.59-9.19) |
|  | Dronedarone | 379 | 7.69(7.11-8.31) | 21 | 5.76(4.24-7.83) | 123 | 6.00(5.27-6.83) | 254 | 17.34(15.80-19.02) | 19 | 1.25(0.91-1.73) |
|  | Ibutilide | 6 | 9.08(3.89-21.19) | 1 | — | 0 | — | 1 | — | 6 | 11.47(4.91-26.78) |
|  | Ivabradine | 757 | 6.99(6.62-7.39) | 72 | 9.49(8.04-11.21) | 350 | 7.82(7.24-8.45) | 234 | 7.37(6.71-8.10) | 222 | 6.60(6.00-7.27) |
|  | Adenosine | 123 | 6.55(5.72-7.5) | 24 | 14.29(10.70-19.10) | 34 | 4.29(3.36-5.48) | 51 | 8.84(7.23-10.82) | 52 | 8.54(6.99-10.42) |
|  | Fluoxetine+Flecainide vs Flecainide | 29 | 1.15(0.85-1.56) | 8 | 2.05(1.22-3.46) | 13 | 1.29(0.85-1.96) | 13 | 1.15(0.76-1.74) | 14 | 2.26(1.50-3.38) |
|  | Duloxetine+Flecainide vs Flecainide | 39 | 0.87(0.68-1.12) | 9 | 1.36(0.84-2.21) | 18 | 1.02(0.72-1.44) | 16 | 0.80(0.55-1.16) | 20 | 1.85(1.32-2.60) |
|  | Paroxetine+Flecainide vs Flecainide | 20 | 0.71(0.50-0.99) | 1 | — | 15 | 1.31(0.89-1.93) | 0 | — | 6 | 0.90(0.50-1.62) |
|  | Amiodarone+Flecainide vs Flecainide | 95 | 1.29(1.08-1.54) | 8 | 0.76(0.46-1.27) | 36 | 1.23(0.96-1.59) | 32 | 0.97(0.74-1.27) | 40 | 2.27(1.77-2.90) |
|  | Citalopram+Propafenone vs Propafenone | 17 | 0.79(0.54-1.16) | 2 | — | 5 | 0.56(0.29-1.07) | 7 | 0.70(0.40-1.22) | 5 | 0.94(0.49-1.81) |
|  | Venlafaxine+Propafenone vs Propafenone | 20 | 1.23(0.85-1.78) | 0 | — | 18 | 2.47(1.68-3.65) | 1 | — | 2 | — |
|  | Sofosbuvir+Amiodarone vs Amiodarone | 66 | 4.10(3.03-5.55) | 16 | 8.69(5.90-12.81) | 35 | 5.87(4.36-7.90) | 31 | 4.58(3.37-6.21) | 18 | 3.20(2.22-4.62) |
|  | Verapamil+Dronedarone vs Dronedarone | 3 | 0.95(0.35-2.00) | 0 | — | 1 | — | 2 | — | 0 | — |
|  | Diltiazem+Dronedarone vs Dronedarone | 27 | 1.96(0.70-1.29) | 1 | — | 4 | 0.46(0.23-0.95) | 23 | 1.21(0.87-1.67) | 1 | — |
|  | Verapamil+Ivabradine vs Ivabradine | 18 | 1.96(1.32-2.89) | 2 | — | 7 | 1.62(0.92-2.84) | 11 | 3.52(2.19-5.64) | 1 | — |
|  | Amiodarone+Ivabradine vs Ivabradine | 76 | 2.01(1.65-2.45) | 9 | 2.34(1.41-3.88) | 19 | 1.09(0.78-1.54) | 32 | 2.69(2.03-3.56) | 36 | 3.17(2.42-4.16) |

Abbreviations: HLGT: high-level group term; HLT: high level term; N: number of records; ROR025: the lower end of the 95% confidence interval of ROR. ROR975: the upper end of the 95% confidence interval of IC; IC025: the lower end of the 95% confidence interval of IC; *p* values was calculated by the chi-square test.

Table 3 Arrhythmia Signal Profiles of Different AAD Strategies.

| P  T | Atrioventricular block complete | | — | — | — | 3.31 | 3.33 | 1.41 | — | 3.15 | 0.77 | — | 1.64 | 2.07 |
| --- | --- | --- | --- | --- | --- | --- | --- | --- | --- | --- | --- | --- | --- | --- |
|  | Atrioventricular block first degree | | — | — | — | 4.88 | 3.71 | 1.47 | — | 2.75 | — | — | -0.08 | — |
|  | Brugada syndrome | | 0.71 | — | — | 4.45 | 0.43 | — | — | 1.17 | — | — | — | — |
|  | Bundle branch block right | | — | — | — | 3.90 | 2.39 | 0.53 | — | 2.23 | — | — | 1.46 | — |
|  | Defect conduction intraventricular | | — | — | — | 4.23 | 1.78 | — | — | 0.89 | — | — | — | — |
|  | Atrioventricular block | | 3.12 | — | — | 2.68 | 2.21 | 2.12 | -1.10 | 3.17 | 2.37 | — | 2.01 | 3.33 |
|  | Atrioventricular block second degree | | — | — | — | 2.10 | — | 1.89 | — | 2.11 | 2.70 | — | 2.20 | — |
|  | Bundle branch block | | — | — | — | 2.54 | — | — | — | 2.91 | — | — | — | — |
|  | Bundle branch block left | | — | — | — | 3.32 | 2.87 | -1.04 | — | 2.95 | 1.08 | — | 3.43 | 0.37 |
|  | Conduction disorder | | — | — | — | 3.69 | 2.40 | — | — | 1.82 | — | — | 1.50 | — |
|  | Long QT syndrome | | — | — | 1.68 | 1.08 | — | 2.76 | — | 3.67 | — | — | — | — |
|  | Sinoatrial block | | — | — | — | 3.29 | — | — | — | 2.71 | — | — | — | — |
|  | Arrhythmia | | 0.44 | 1.63 | 1.31 | 3.58 | 3.58 | 2.92 | 3.21 | 2.68 | 2.75 | — | 1.58 | -1.43 |
|  | Bradyarrhythmia | | — | — | — | 4.08 | 2.85 | — | — | 3.02 | 2.20 | — | — | — |
|  | BRASH syndrome | | — | — | — | — | — | 3.05 | — | 4.26 | — | — | — | — |
|  | Cardiac flutter | | — | — | — | 2.81 | 2.60 | 1.84 | 2.24 | 0.72 | 1.67 | — | — | — |
|  | Tachyarrhythmia | | — | — | — | 4.62 | 0.72 | — | 0.49 | 4.59 | — | — | 1.20 | — |
|  | Bradycardia | | 0.31 | 1.22 | — | 3.81 | 3.83 | 3.00 | -0.29 | 3.63 | 1.71 | — | 3.67 | 1.43 |
|  | Cardiac fibrillation | | — | — | — | 1.39 | 2.19 | 0.49 | 0.16 | 1.65 | 0.38 | — | 0.71 | — |
|  | Extrasystoles | | — | — | — | 1.77 | 2.47 | 0.76 | 2.71 | 1.99 | 0.70 | — | 1.17 | — |
|  | Tachycardia | | — | — | -0.61 | 1.99 | 1.34 | 0.69 | 1.19 | 1.21 | 0.98 | — | 1.97 | 0.57 |
|  | Arrhythmia supraventricular | | — | — | — | 1.98 | 1.62 | 0.10 | 1.01 | 2.27 | — | — | 1.81 | — |
|  | Atrial fibrillation | | — | 1.56 | -1.91 | 4.04 | 3.84 | 3.65 | 4.40 | 3.50 | 4.27 | — | 1.48 | 1.34 |
|  | Nodal arrhythmia | | — | — | — | 1.44 | 3.12 | — | — | 3.48 | — | — | — | — |
|  | Sinus arrest | | — | — | — | 1.74 | 1.89 | 1.72 | — | 1.56 | — | — | 2.44 | 0.60 |
|  | Sinus bradycardia | | — | — | — | 3.28 | 4.09 | 2.61 | 0.21 | 3.66 | -0.76 | — | 3.85 | — |
|  | Sinus tachycardia | | — | — | — | -0.01 | — | -1.26 | — | -0.67 | — | — | 3.03 | -0.22 |
|  | Atrial flutter | | — | — | — | 4.53 | 3.7 | 3.76 | 3.69 | 3.94 | 3.12 | — | 1.12 | — |
|  | Atrial tachycardia | | — | — | — | 2.81 | — | 2.09 | 1.95 | 4.41 | — | — | 4.08 | — |
|  | Nodal rhythm | | — | — | — | 0.00 | 1.07 | — | — | 2.50 | — | — | — | — |
|  | Sinus arrhythmia | | — | — | — | — | — | — | 1.49 | 1.65 | 0.49 | — | — | — |
|  | Sinus node dysfunction | | — | — | — | 3.50 | — | 1.50 | 0.98 | 4.05 | 0.15 | — | 2.73 | — |
|  | Supraventricular extrasystoles | | — | — | — | 2.14 | -1.81 | 1.08 | 2.77 | 3.28 | — | — | 3.21 | — |
|  | Supraventricular tachycardia | | — | — | — | 3.06 | 2.61 | 1.16 | 1.34 | 1.81 | — | — | 0.65 | 4.50 |
|  | Cardiac arrest | | — | 1.74 | -1.55 | 2.66 | 2.36 | 1.37 | 2.00 | 2.24 | -1.68 | — | 1.29 | 1.26 |
|  | Sudden death | | — | — | — | — | — | 0.65 | — | 1.58 | — | — | 2.45 | — |
|  | Ventricular arrhythmia | | — | 0.52 | 2.81 | 2.73 | — | 2.71 | 0.07 | 3.73 | — | — | 3.16 | — |
|  | Ventricular extrasystoles | | — | — | 2.60 | 2.14 | — | 1.08 | 2.77 | 3.28 | 0.04 | — | 3.21 | 0.73 |
|  | Ventricular tachycardia | | 1.05 | 1.29 | 3.90 | 4.35 | 4.12 | 3.49 | 3.92 | 4.57 | -1.17 | — | 3.43 | 3.39 |
|  | Cardio-respiratory arrest | | — | 0.62 | — | 1.98 | 2.45 | 1.63 | -1.92 | 2.10 | — | — | 1.64 | -1.19 |
|  | Pulseless electrical activity | | — | — | — | 3.59 | 0.26 | — | — | 1.92 | — | — | 1.19 | — |
|  | Torsade de pointes | | — | — | 1.80 | 3.31 | — | 4.84 | 4.51 | 4.93 | — | 1.89 | 4.35 | — |
|  | Ventricular fibrillation | | — | 2.18 | 1.33 | 3.93 | 2.00 | 1.85 | 3.07 | 4.34 | -0.75 | — | 3.26 | 3.67 |
|  | IC025≤0 |  | Quinidine | Disopyramide | Mexiletine | Flecainide | Propafenone | Sotalol | Dofetilide | Amiodarone | Dronedarone | Ibutilide | Ivabradine | Adenosine |
|  | 4 >IC025>0 |  |  |  |  |  |  |  |  |  |  |  |  |  |
|  | IC025≥4 | 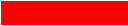 |  |  |  |  |  |  |  |  |  |  |  |  |

Table 4 Onset time of AADs–associated arrhythmias.

|  | Median (IQR) | 0-30 | 31-60 | 61-90 | 91-120 | 121-180 | 181-360 | Greater than 360 | Unknown |
| --- | --- | --- | --- | --- | --- | --- | --- | --- | --- |
| Quinidine (n=27) | -- | 0(0.00%) | 0(0.00%) | 0(0.00%) | 0(0.00%) | 0(0.00%) | 0(0.00%) | 0(0.00%) | 27(100.00%) |
| Disopyramide (n=54) | 13 (0-84) | 10(18.52%) | 1(1.85%) | 0(0.00%) | 0(0.00%) | 0(0.00%) | 1(1.85%) | 2(3.70%) | 40(74.07%) |
| Mexiletine (n=65) | 11 (1-139) | 12(18.46%) | 0(0.00%) | 1(1.54%) | 1(1.54%) | 1(1.54%) | 1(1.54%) | 3(4.62%) | 46(70.77%) |
| Flecainide (n=1675) | 47(4-349) | 216(12.90%) | 37(2.21%) | 23(1.37%) | 10(0.60%) | 21(1.25%) | 50(2.99%) | 118(7.04%) | 1200(71.64%) |
| Propafenone (n=644) | 112(3-433) | 63(9.78%) | 7(1.09%) | 5(0.78%) | 10(1.55%) | 9(1.40%) | 20(3.11%) | 47(7.30%) | 483(75.00%) |
| Sotalol (n=1179) | 64 (3-351) | 181(15.35%) | 22(1.87%) | 29(2.46%) | 22(1.87%) | 16(1.36%) | 41(3.48%) | 103(8.74%) | 765(64.89%) |
| Dofetilide (n=778) | 43 (2-332) | 128(16.45%) | 18(2.31%) | 11(1.41%) | 12(1.54%) | 15(1.93%) | 23(2.96%) | 66(8.48%) | 505(64.91%) |
| Amiodarone (n=5657) | 46 (5-330) | 864(15.27%) | 141(2.49%) | 97(1.71%) | 38(0.67%) | 101(1.79%) | 219(3.87%) | 446(7.88%) | 3751(66.31%) |
| Dronedarone (n=379) | 165(14-565) | 29(7.65%) | 9(2.37%) | 3(0.79%) | 3(0.79%) | 8(2.11%) | 11(2.90%) | 36(9.50%) | 280(73.89%) |
| Ibutilide (n=6) | 0(0-0) | 2(33.33%) | 0(0.00%) | 0(0.00%) | 0(0.00%) | 0(0.00%) | 0(0.00%) | 0(0.00%) | 4(66.67%) |
| Ivabradine (n=757) | 14 (0-132) | 135(17.83%) | 17(2.25%) | 13(1.72%) | 9(1.19%) | 13(1.72%) | 12(1.59%) | 39(5.15%) | 519(68.56%) |
| Adenosine (n=123) | 65 (0-366) | 11(8.94%) | 1(0.81%) | 4(3.25%) | 1(0.81%) | 2(1.63%) | 1(0.81%) | 6(4.88%) | 97(78.86%) |
| Fluoxetine/Duloxetine/Paroxetine/Amiodarone + Flecainide (n=183) | 18(2-245) | 26(14.21%) | 1(0.55%) | 1(0.55%) | 0(0.00%) | 2(1.09%) | 5(2.73%) | 7(3.83%) | 140(76.50%) |
| Citalopram/Venlafaxine + Propafenone (n=37) | 19 (12-474) | 5(13.51%) | 1(2.70%) | 0(0.00%) | 0(0.00%) | 0(0.00%) | 0(0.00%) | 3(8.11%) | 28(75.68%) |
| Sofosbuvir + Amiodarone (n=66) | 18 (0-81) | 12(18.18%) | 2(3.03%) | 4(6.06%) | 0(0.00%) | 0(0.00%) | 0(0.00%) | 5(7.58%) | 43(65.15%) |
| Verapamil/Diltiazem + Dronedarone (n=30 ) | 325 (1-668) | 3(10.00%) | 1(3.33%) | 0(0.00%) | 0(0.00%) | 0(0.00%) | 2(6.67%) | 5(16.67%) | 19(63.33%) |
| Verapamil/Amiodarone + Ivabradine (n=94) | 0 (0-146) | 18(19.15%) | 1(1.06%) | 1(1.06%) | 1(1.06%) | 2(2.13%) | 1(1.06%) | 5(5.32%) | 65(69.15%) |

Abbreviations: N: number of records; IQR: interquartile range; AAD：antiarrhythmic drug.

**Table S1. Table with “Cardiac arrhythmias” and PTs used.**

| Arrhythmias in HLT level | Arrhythmias in PT level |
| --- | --- |
| Cardiac conduction disorders | Atrioventricular block complete |
|  | Atrioventricular block first degree |
|  | Brugada syndrome |
|  | Bundle branch block right |
|  | Defect conduction intraventricular |
|  | Atrioventricular block |
|  | Atrioventricular block second degree |
|  | Bundle branch block |
|  | Bundle branch block left |
|  | Conduction disorder |
|  | Long QT syndrome |
|  | Sinoatrial block |
| Rate and rhythm disorders NEC | Arrhythmia |
|  | Bradyarrhythmia |
|  | BRASH syndrome |
|  | Cardiac flutter |
|  | Tachyarrhythmia |
|  | Bradycardia |
|  | Cardiac fibrillation |
|  | Extrasystoles |
|  | Tachycardia |
| Supraventricular arrhythmias | Arrhythmia supraventricular |
|  | Atrial fibrillation |
|  | Nodal arrhythmia |
|  | Sinus arrest |
|  | Sinus bradycardia |
|  | Sinus tachycardia |
|  | Atrial flutter |
|  | Atrial tachycardia |
|  | Nodal rhythm |
|  | Sinus arrhythmia |
|  | Sinus node dysfunction |
|  | Supraventricular extrasystoles |
|  | Supraventricular tachycardia |
| Ventricular arrhythmias and cardiac arrest | Cardiac arrest |
|  | Sudden death |
|  | Ventricular arrhythmia |
|  | Ventricular extrasystoles |
|  | Ventricular tachycardia |
|  | Cardio-respiratory arrest |
|  | Pulseless electrical activity |
|  | Torsade de pointes |
|  | Ventricular fibrillation |

Abbreviations: HLT: high level term; PT: preferred term.
